# Peripheral Ossifying Fibroma Evolved From Pyogenic Granuloma

**DOI:** 10.7759/cureus.20904

**Published:** 2022-01-03

**Authors:** Géssica V Godinho, Cristhiane A Silva, Bruno R Noronha, Everton J Silva, Luiz E Volpato

**Affiliations:** 1 Department of Dentistry, Hospital de Câncer de Mato Grosso, Cuiabá, BRA; 2 Program on Environment and Health, Universidade de Cuiabá, Cuiabá, BRA; 3 Cuiabá Dental School, Universidade de Cuiabá, Cuiabá, BRA

**Keywords:** gingival growth, excisional tissue biopsy, pyogenic granuloma, ossifying fibroma, gingival neoplasms

## Abstract

The aim of the present article is to present the clinical case of a large peripheral ossifying fibroma that evolved from a previously diagnosed pyogenic granuloma in a 50-year-old woman. The patient was referred for treatment of a lesion over the buccal and palatal gingiva close to the left upper first molar. It was purplish-red in color, approximately 3 cm in diameter, having a smooth surface, a pedicled and bleeding base, with seven years of evolution, and diagnosed as pyogenic granuloma. After three years of evasion, the patient returned reporting an increase in the lesion and difficulty in eating. Clinically the nodule was lobular in appearance, pink in color and smooth, pediculated, firm in consistency, non-bleeding, about 5 cm in its greatest extension, extending to the maxillary tuberosity. The lesion was excised and referred for histopathological examination, which led to the diagnosis of peripheral ossifying fibroma. The patient was followed for approximately 18 months, prosthetically rehabilitated, with satisfactory healing and no clinical signs of recurrence.

The possible evolution of a pyogenic granuloma to a peripheral ossifying fibroma was observed in this case, based on the histopathological changes that occurred, with the development of calcified material, fibrous maturation, and decreased vascular content of the initial lesion after three years.

## Introduction

Pyogenic granuloma (PG) is the second most frequent reactive lesion of the oral cavity, after inflammatory fibrous hyperplasia, the most frequent reactive lesion. PG presents a well-circumscribed and distinct lobular arrangement, with large central vessels and well-formed peripheral capillary aggregates. When left untreated, this lesion can undergo fibrous maturation with ossification and develop into peripheral ossifying fibroma [1].

Peripheral ossifying fibroma (POF) is a benign mesenchymal lesion, predominant in the anterior maxillary gingiva of women between the second and third decades of life. It is considered a metaplastic process that involves the periodontal ligament in response to trauma or local irritants, such as biofilm, calculus, poorly adapted prostheses, and unsatisfactory restorations [2,3]. The evolution from PG to POF is explained due to their clinical and histopathological similarities; however, it is important to note that not all POF evolves from a PG. The mineralized component formed can vary among cementum, bone, and dystrophic calcification [4].

This report presents the case of a large peripheral ossifying fibroma that evolved from a lesion previously diagnosed as pyogenic granuloma in a 50-year-old woman.

## Case presentation

**Figure 1:**
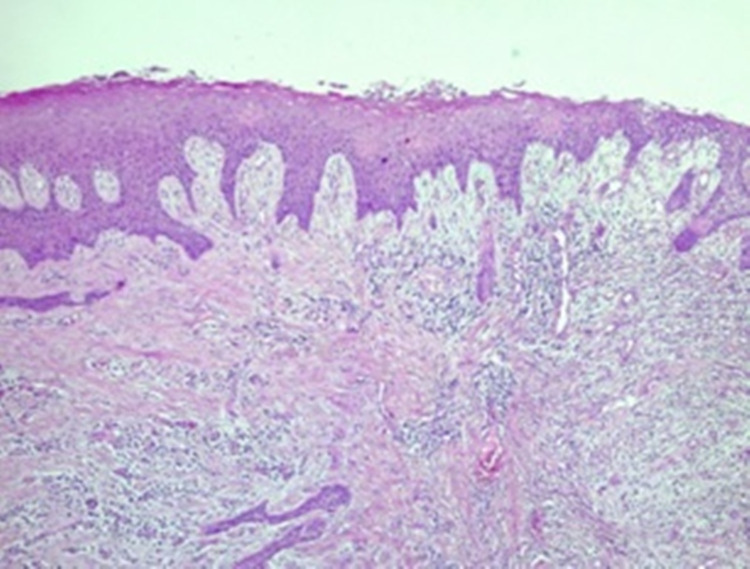
Histological image of pyogenic granuloma showing hyperparakeratinized stratified squamous epithelium, with areas of acanthosis and long projections. Lamina propria formed by dense connective tissue, noting areas of intense cellularity and proliferation of endothelial cells, with opening of vascular spaces and regions of intense chronic inflammatory infiltrate with hemorrhagic areas (Haemotoxylin and Eosin, 100x).

A 50-year-old hypertensive woman was referred to the Oral and Maxillofacial Surgery and Traumatology outpatient clinic of the Mato Grosso Cancer Hospital, Cuiabá, Brazil, for treatment of a maxillary lesion, diagnosed as PG, as demonstrated by the histopathological examination of an incisional biopsy performed previously. The microscopic description consisted of a fragment of mucosa covered by stratified hyperparakeratinized squamous epithelium, with areas of acanthosis and long projections, spongiosis, exocytosis, and ulceration. The lamina propria was formed by dense connective tissue, with areas of intense cellularity and proliferation of endothelial cells being noted, with opening of vascular spaces and regions of intense chronic inflammatory infiltrate with hemorrhagic areas (Figure 1).

Clinically, she presented a nodule over the buccal and palatal gingiva close to the left upper first molar, approximately 3 cm in diameter, with a smooth surface, purplish-red color, pedicled and bleeding base with seven years of evolution. In the referral, there was also the diagnosis of severe chronic periodontitis, observing, during the intraoral physical examination, poor oral hygiene and generalized tooth mobility.

Due to the size and potential bleeding, in addition to the patient's medical history, it was decided to perform the excision of the lesion under general anesthesia, and to extract all teeth due to the degree of mobility and loss of bone insertion, aiming at further rehabilitation with total prosthesis.

**Figure 2:**
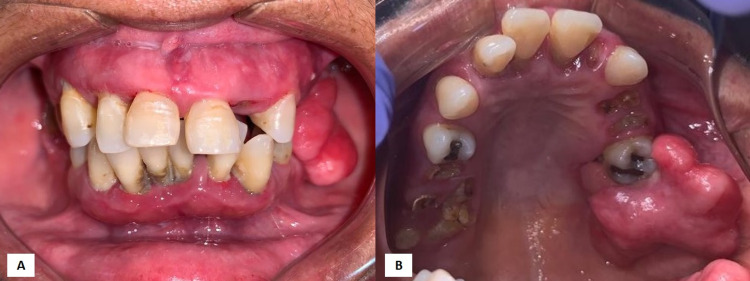
Intraoral images. (A) Front view showing vertical extension of the lesion, reaching the posterior region of the mandible. Unsatisfactory oral hygiene, non-healthy teeth and hyperemic gingiva are also observed; (B) Occlusal view showing a volumetric increase in the posterior margin of the maxilla on the left involving the buccal and palatal regions, extending from the first molar to the maxillary tuberosity. Poor oral hygiene, presence of calculus, periodontal infection and residual roots are also observed.

After three years of evasion, the patient returned reporting an increase in the lesion and difficulty in eating due to the trauma generated during chewing. Upon clinical examination, the nodule was lobular in appearance, pink in color and smooth, pediculated, firm in consistency, non-bleeding, about 5 cm in its greatest extension, inserted in the buccal and palatal gingival region of the left upper first molar, extending to the maxillary tuberosity. By this time, the duration of the symptoms was about 10 years (Figure 2).

**Figure 3:**
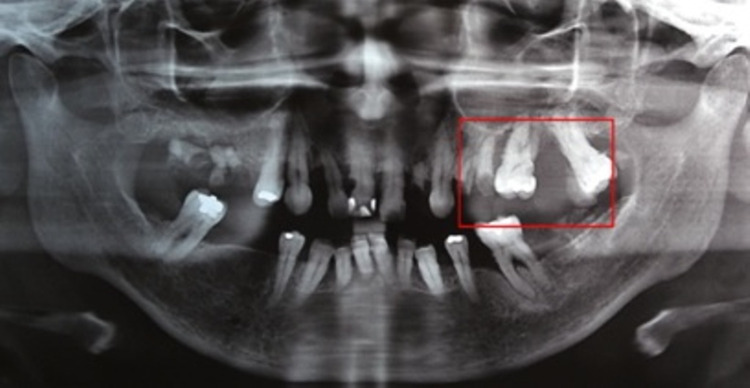
Panoramic radiograph showing a radiopaque image associated with the left posterior maxillary alveolar process, with divergence of the crowns of the first and second molars and loss of bone insertion.

The panoramic radiograph showed, in the area of the lesion, diffuse irregular radiopacity, with divergence of the crowns of the first and second molars, in addition to generalized loss of bone insertion (Figure 3).

**Figure 4:**
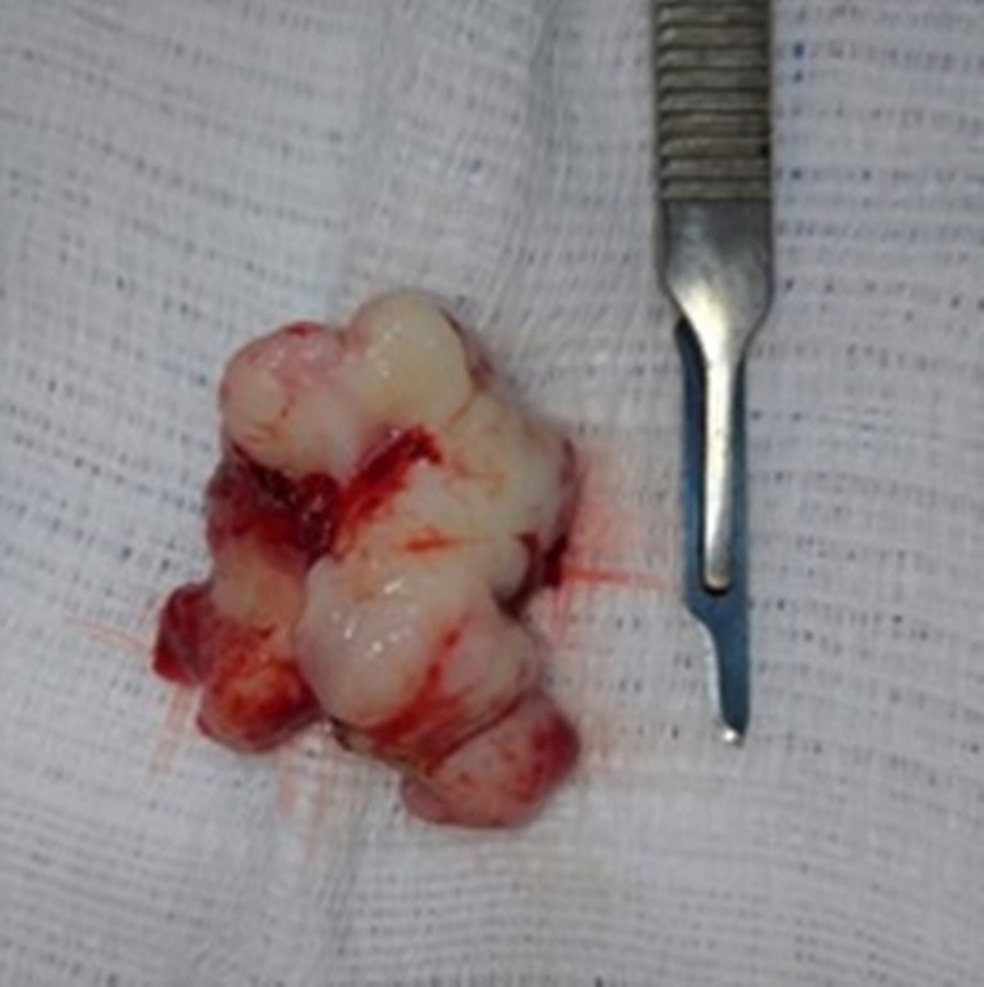
Macroscopic image of the excised lesion, showing an irregular aspect and lobulated surface.

The lesion was excised and the teeth were extracted under general anesthesia. The lesion, measuring approximately 5x4.5x3cm, was referred for histopathological examination, which led to the diagnosis of POF (Figure 4).

**Figure 5:**
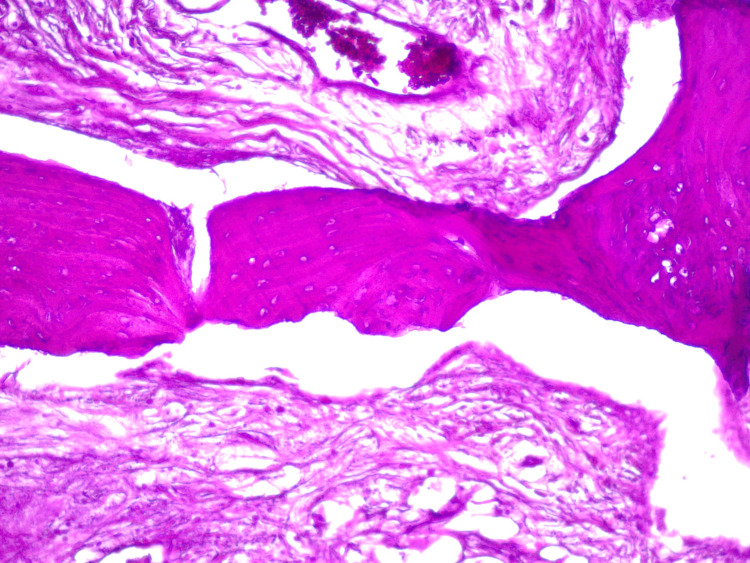
Histological image of peripheral ossifying fibroma showing dense connective tissue with intense cellularity, formation of osteoid material, and inflammatory infiltrate (H&E, 400x). H&E: Haemotoxylin and Eosin

The histopathological report of the excised lesion revealed a fragment of mucosa covered by stratified keratinized squamous epithelium with long projections, exocytosis, and spongiosis. In the lamina propria formed by dense connective tissue, intense cellularity was noted, with elongated cells, formation of osteoid material, and intense mononuclear inflammatory infiltrate, with diagnosis of POF (Figure 5).

**Figure 6:**
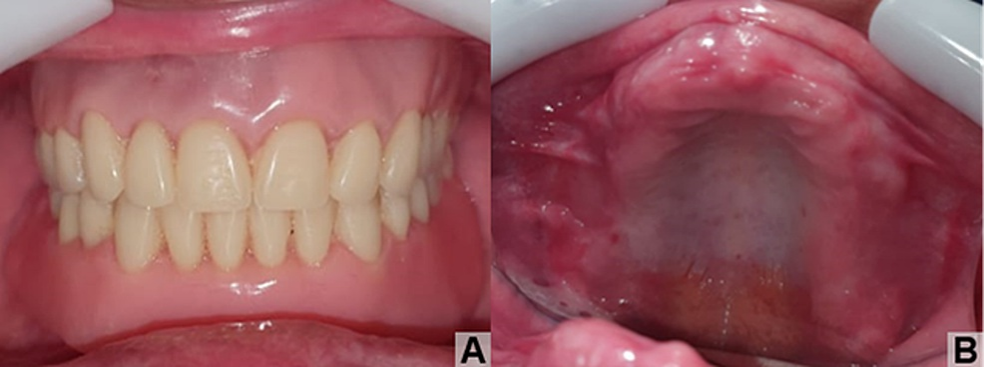
(A) Rehabilitated patient with superior and inferior total prostheses; (B) Clinical aspect in occlusal view of the maxilla, showing healthy and normocorated mucous membranes, with absence of pathological changes.

The patient has been followed for approximately 18 months, prosthetically rehabilitated with satisfactory healing and no clinical signs of recurrence (Figure 6).

## Discussion

We presented the case of a POF that had been diagnosed three years earlier as PG. POF and PG are reactive lesions of the oral cavity developed from low-intensity chronic irritation, stimulating an intense tissue repair response and producing increases in soft tissue volume [5].

Clinically, the lesions have some distinctions: a POF is generally firm, pale pink to dark red, while a PG has a soft consistency, ranging from dark red to reddish purple, with a tendency to bleed easily [6,7]. As reported, the PG lesion had mixed color and bleeding aspect, while the POF was pink and not bleeding. These differences can be explained by the histological aspect of POF, which has keratinized stratified squamous epithelium with hyperkeratosis, acanthosis, pseudoepitheliomatous hyperplasia, and less pronounced inflammatory infiltrates, in which the stroma is mainly collagenized, contrasting with PG, which consists mainly of tissue proliferation granulation, capillaries, venules, lobes of vascular neoformations, and marked inflammatory infiltrates [6]. In the microscopic descriptions presented in this report, the presence of intense cellularity and proliferation of endothelial cells is observed in addition to hemorrhagic areas in the diagnosis of PG; this characteristic no longer exists in the description of POF.

Due to their clinical characteristics and common etiological factors, clinical differentiation between the lesions is difficult, requiring microscopic evaluation for a definitive diagnosis [6]. Histopathologically, the diagnosis of POF includes criteria such as the presence of fibrous connective tissue with variable fibroblast, myofibroblast and collagen content, scarce to abundant endothelial proliferation, and mineralized material. These mineralized components vary from 23-75% and can be of three types: bone, dystrophic calcifications, or cementum [7,8]. A study demonstrated the presence of oxitan fibers around the mineralized components of the POF and a larger area occupied by the connective tissue stroma in relation to the mineralized components when compared to other fibro-bone lesions, in addition to a higher collagen density [9]. In histological terms, POF is more cellular and less vascular than PG [6]. In the case presented, the two histopathological exams differed basically due to the absence of vascularized content and the presence of calcified material in the material collected later, which led to the diagnosis of POF.

As discussed previously, the expression of osteopontin, a non-collagenous protein with high calcium binding potential, influencing tissue mineralization, is totally absent in normal oral mucosa, for example, but in some cases of PG there is expression in stromal cells and extracellular matrix, favoring the concept that PG can mature into POF. In comparison, there is expression of osteopontin in stromal cells, extracellular matrix, and in areas of ossification in all cases of POF [10]. It is suggested that PG may evolve to POF; however, it is also possible to find POF as the primary diagnosis, with the PG as a recurrent lesion [3]. This interrelation was observed in this case, in which a lesion submitted to incisional biopsy was diagnosed as PG but was not treated and, three years later, the lesion was submitted to a new histopathological exam, the result of which was a POF, supporting the hypothesis that they may represent different stages of the same pathological entity. Although the interrelationship between the two lesions has been previously reported [3,6], following the maturation process and histopathological evolution of a single lesion is rare and no other case report similar to this one has been found.

The presence of trauma or local irritants to cells of the periodontal ligament is identified as the etiology of POF. The occurrence exclusively in the gingiva, the proximity of the gingiva to the periodontal ligament, and the occurrence of oxitan fibers in the mineralized matrix support this etiological hypothesis [2,4]. In the case presented, the patient had unsatisfactory oral hygiene, suggesting that biofilm and dental calculus were the main irritating factors to the periodontal ligament.

POF is prevalent in young patients and has a predilection for the female sex [11]; however, in this case, the lesion developed in an adult patient, between the fourth and fifth decades of life, presenting with an unusual extensive size of 5 cm. Migration of teeth adjacent to the POF is uncommon; however, it happens in some cases [4], as also seen in our case.

Surgical excision is the treatment of choice, with peripheral and deep margins, in addition to the removal of the etiological agent. In gingival lesions, curettage of the underlying tissue is recommended. The recurrence of reactive lesions of the oral cavity is about 20% and is directly related to the failures in these maneuvers [1,2,12]. The treatment performed consisted of excision of the lesion with adequate margins and extraction of all teeth, since the patient was also diagnosed with severe chronic periodontitis. Attention was given to the instruction of oral hygiene and hygiene of prostheses made after surgical treatment, alerting the patient about the influence of these factors on the etiology of the lesions presented.

## Conclusions

The possible evolution of a PG to a POF was observed in this case based on the histopathological changes that occurred, with the development of calcified material, fibrous maturation, and decreased vascular content of the initial lesion after three years. Considering the reactive nature of the two lesions and biofilm and calculus as local irritants, oral hygiene guidance is as important as the recommended surgical treatment, thus minimizing the risk of recurrence.
